# Detecting intention to walk in stroke patients from pre-movement EEG correlates

**DOI:** 10.1186/s12984-015-0087-4

**Published:** 2015-12-12

**Authors:** Andreea Ioana Sburlea, Luis Montesano, Roberto Cano de la Cuerda, Isabel Maria Alguacil Diego, Juan Carlos Miangolarra-Page, Javier Minguez

**Affiliations:** Bit & Brain Technologies S.L., Calle Maria de Luna 11, nave 4, Zaragoza, 50018 Spain; University of Zaragoza, Institute of Investigation in Engineering of Aragon (I3A), Building I+D+i, Mariano Esquillor, Zaragoza, 50018 Spain; Department of Physiotherapy, Occupational therapy, Rehabilitation and Physical Medicine, Faculty of Health Sciences, Alcorcon, Madrid Spain

**Keywords:** EEG, Stroke, Gait rehabilitation, BCI, MRCP, ERD

## Abstract

**Background:**

Most studies in the field of brain-computer interfacing (BCI) for lower limbs rehabilitation are carried out with healthy subjects, even though insights gained from healthy populations may not generalize to patients in need of a BCI.

**Methods:**

We investigate the ability of a BCI to detect the intention to walk in stroke patients from pre-movement EEG correlates. Moreover, we also investigated how the motivation of the patients to execute a task related to the rehabilitation therapy affects the BCI accuracy. Nine chronic stroke patients performed a self-initiated walking task during three sessions, with an intersession interval of one week.

**Results:**

Using a decoder that combines temporal and spectral sparse classifiers we detected pre-movement state with an accuracy of 64 % in a range between 18 % and 85.2 %, with the chance level at 4 %. Furthermore, we found a significantly strong positive correlation (*r* = 0.561, *p* = 0.048) between the motivation of the patients to perform the rehabilitation related task and the accuracy of the BCI detector of their intention to walk.

**Conclusions:**

We show that a detector based on temporal and spectral features can be used to classify pre-movement state in stroke patients. Additionally, we found that patients’ motivation to perform the task showed a strong correlation to the attained detection rate of their walking intention.

## Introduction

Stroke is one of the leading causes of neurological disability among adults and often causes movement impairments [1]. For patients with motor deficits in lower extremities, gait rehabilitation can improve activities of daily living [2]. A new tendency in rehabilitation is to involve not only the patient’s body but also their brain. This tendency, called “human in the loop”, allows to increase the engagement and motivation of the patients while actively performing the tasks [3]. It has been shown that cortical plasticity of both brain hemispheres contributes to successful gait rehabilitation [4–6]. In order to facilitate closing the loop, rehabilitation devices should be able to detect cognitive processes related to the therapy. This can be achieved by using electroencephalography (EEG) based brain-computer interface (BCI).

One of the cognitive processes involved in rehabilitation is the intention to move. Neural correlates of movement intention, as measured by EEG, are the motor-related cortical potential (MRCP) [7, 8] and the event-related (de)synchronization (ERD/S) [9]. Since these correlates anticipate movement, they can be used to trigger prosthetic devices such as exoskeletons, and facilitate the usage of BCIs in rehabilitation [10–13].

Lower-limbs pre-movement state has been recently studied in healthy subjects when performing movements such as ankle dorsiflexion [14], knee movements [15], simultaneous dorsiflexion of both feet [16], sitting-standing transitions [17] or analysis of lower limbs in the context of gait function [18–22]. However, only few studies have shown the feasibility of these methods on stroke patients [23, 24]. In one of the studies [23], MRCPs are used for the detection of pre-movement states when performing overt and covert ankle-dorsiflexion in both healthy and stroke subjects. Another study [24] relies on the detection of ERD as a pre-movement correlate when performing ankle-dorsiflexions, for the control of a functional electrical stimulation (FES) rehabilitation device. To our knowledge, this is the first study about the detection of self-initiated walking, relying on EEG correlates, in stroke patients.

On the basis of an experimental setup with nine stroke patients that underwent three recording sessions with one week between them, we address two hypotheses. First, based on a previous study with healthy subjects [22], we hypothesize that an EEG-based decoder that combines temporal and spectral features can detect the pre-movement state of stroke patients. Next, we surmise that the intrinsic-motivation of the patients when performing tasks related to gait-rehabilitation can positively modulate the accuracy of the EEG-based detector.

## Materials and methods

### Experimental procedure

Nine chronic stroke patients (three females, mean age = 59.7 years, SD = 11.3 years) participated in the experiment. They were recruited from the Universitary Clinic of University Rey Juan Carlos in Madrid, Spain. Demographic and clinical information of the participants is shown in Table 1. All patients suffered hemispheric stroke (four on the right side and five on the left side). Three of the patients suffered a hemorrhagic stroke and six an ischemic stroke. According to the Waterloo Footedness Questionnaire [25] four out of nine subjects were right-footed. The Fugl-Meyer Assessment for Lower Extremity (FMA-LE), which consists of 43 items, with a maximum possible score of 86 points was administered to all patients. Each item was answered using a 3-point ordinal scale (0 = cannot perform, 1 = can partially perform, 2 = can fully perform). The assessment was completed by trained registered physical therapists. The experimental protocol was approved by the ethical committee of the HYPER project (approval number 12/104)^1^ and all patients gave written consent before participating in the experiment.

**Table 1:** Demographic and clinical information of nine stroke patients

Patient	Gender	Age	Type	Affected brain	Time since	Fugl-Meyer	Particular
number		(years)	of ictus	hemisphere	ictus (years)	score	brain condition
1	F	52	ischemic	left	4.75	86	no
2	F	54	ischemic	right	16.5	66	no
3	M	41	hemorrhagic	right	13.66	62	brain surgery
4	F	69	hemorrhagic	left	2.33	81	no
5	M	76	hemorrhagic	left	1.75	73	no
6	M	67	ischemic	left	9.75	80	no
7	M	54	ischemic	right	4.66	69	titanium plate
8	M	71	ischemic	left	2	55	no
9	M	54	ischemic	right	1.42	72	no

**Fig. 1:**
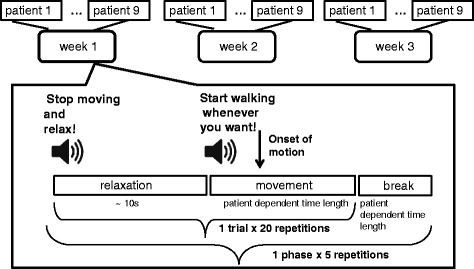
Protocol of the experiment

The experiment took place in the Motion Analysis, Ergonomics, Biomechanics and Motor Control Laboratory (LAMBECOM), Faculty of Health Sciences, Rey Juan Carlos University, Madrid, Spain. Patients performed the experiment in three sessions, with a week between sessions. Figure 1 illustrates the protocol of the experiment. The experimental protocol was the same during all three sessions. Each trial was composed of two parts: relaxation and movement. Both parts had variable time lengths according to the patient needs. The relaxation part started with an auditory cue that instructed the patients to relax and reduce movements. After approximately ten seconds another auditory cue instructed the patients to start walking whenever they want. Patients were previously instructed to wait a couple of seconds after hearing the second auditory cue. After every twenty trials there were break intervals with a duration adjusted to the need of the patients. A phase consisted of twenty trials and a break interval. A session had five phases, comprising a total of 100 trials.

### Data acquisition and preprocessing

EEG data were recorded using a TMSi Refa amplifier and a 30-channel EEG cap prepared for water based electrodes (from TMSi, Enschede, The Netherlands). The EEG sensors were located at Fp1, Fpz, Fp2, F7, F3, Fz, F4, F8, FC5, FC1, FC2, FC6, T7, C3, Cz, C4, T8, CP5, CP1, CP2, CP6, P7, P3, Pz, P4, P8, POz, O1, Oz, O2, following the 10/10 international system, with the ground placed on the right wrist and two sensors on the ear lobes used for average linked ears reference. Electromyographic (EMG) data were recorded with the same amplifier using two bipolar Ag/AgCl electrodes on the top of tibialis anterior muscles of the right and left legs. This muscle was chosen as being consistently reported as one of the first that activate in walking [26] of healthy subjects. EEG and EMG electrodes impedance was kept below 5 *KΩ*, and below 20 *KΩ*, respectively. Shielded cables (TAS2, TMSi) were used to diminish the artifacts introduced by cable movements. EEG and EMG signals were recorded at a sampling frequency of 256 Hz, without filtering. The amplifier was carried by the subjects in a backpack during the experiment.

**Fig. 2:**
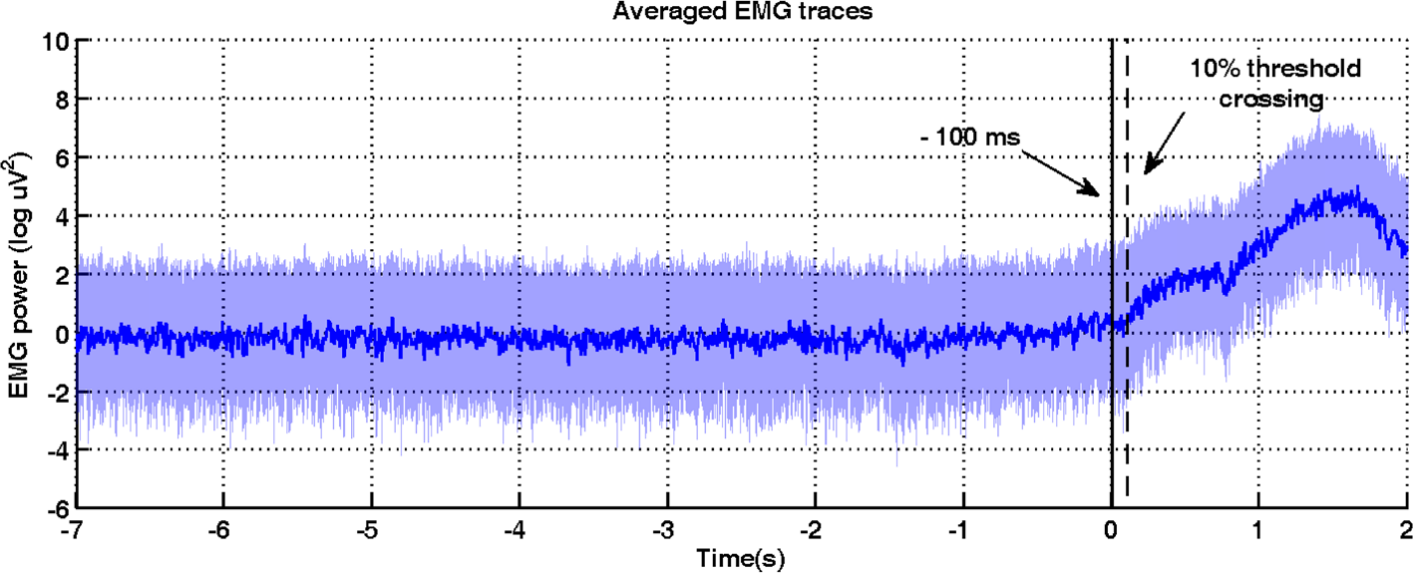
EMG traces of tibialis anterior muscles of the right or left leg for one subject during one recording session

EEG and EMG data preprocessing follows the methodology in [22]. For the computation of movement onset, EMG data were segmented in trials between the relaxation cue and five seconds after the preparation of movement cue. Next, the data was Hilbert transformed and a threshold of 10 % of the highest value was computed. The onset of motion was calculated as 100 ms before the considered threshold crossing. Figure 2 presents on a logarithmic scale, the power of EMG traces aligned to the movement onset, for one representative subject during one recording session.

Trials in which the onset of motion was detected before the preparation of movement cue were rejected as considered artifactual due to erroneous execution of the experimental protocol. After trial rejection, there were in average across subjects and sessions 96 remaining trials, in a range between 86 and 100 trials. Segments of EEG data (hereafter called trials) with a length of six seconds were extracted prior to the movement onset.

### Neurophysiological analysis

On the remaining trials, artifacts were removed in two steps. First, within each EEG channel a trial exclusion criteria was imposed by improbable data appearance. This exclusion criteria was computed using the joint probability function of EEGLAB 13.3.2 toolbox [27]. The trials that were outside three standard deviations of the estimated probability distribution per channel were rejected. Next, EEG data were processed with FastICA [28] and the independent components with an amplitude larger than two times the standard deviation of the mean values were rejected as considered artifactual. The remaining components were projected back to the sensor-space for the rest of the analysis.

Movement related cortical potentials and event-related desynchronization were used to assess pre-movement cortical activity. For the MRCP analysis, EEG data were band-pass filtered with a zero-phase shift Butterworth second order filter at 0.1−1 Hz and downsampled to 10 Hz. For the ERD, the time-frequency representation of the power was computed using Morlet wavelets [29], with a resolution of 0.5 Hz in the frequency band 0.1−30 Hz. The significant ERD maps were computed using a baseline interval between −4 to −2 s with a bootstrap analysis at a significance level of *p*= 0.05 [30]. sLORETA [31] was used to visualize the estimated location of MRCP and ERD activity at the onset of motion using the default parameters (no regularization).

### Feature extraction and classification

Following a similar feature extraction procedure to the one described in [22], we extracted features from ten channels (F3, Fz, F4, FC1, FC2, C3, Cz, C4, CP1, CP2) located over the precentral, central and postcentral motor and sensorimotor cortex. The MRCP features were the amplitudes of the signal in the 0.1−1 Hz frequency band. The ERD features were the logarithmic power of the signals in the *μ* (8−13 Hz) frequency band. Features for both processes were extracted with a one second long sliding window in steps of 125 ms. Windows previous to −1.5 s time moment (w.r.t. the movement onset) were labeled ‘relaxation state’ and those following −1.5 s time moment, were labeled ‘pre-movement state’. We extracted a total of 41 windows out of which 8 belonged to the pre-movement class.

For model selection and classification we used sparse linear discriminant analysis (SLDA) [32]. The SLDA regularization hyperparameters (*ℓ*_1_ and an *ℓ*_2_-norm) were selected in the five-fold inner cross-validation loop. We used a detection model on two layers for the classification of pre-movement state. The first layer has two single-layer detection models based on MRCP and ERD features, while in the second layer we used linear discriminant analysis. A more detailed description of the two-layered model can be found in [22].

### Detection model evaluation

On the trials retained after data preprocessing, we performed classification analysis in a 5 × 5-fold nested chronological cross-validation [33]. The nested cross-validation contained an outer and an inner loop. In the outer loop, chronologically ordered trials were separated into training and test folds. On the training folds of the outer loop, we rejected artifactual trials using joint probability, as described in the Neurophysiological analysis subsection, and learned the parameters of FastICA. The features of the training folds were normalized to Euclidean length. On the training folds we ran the inner loop of the cross-validation (model selection), in which we selected the SLDA hyperparameters in a grid search and the probability threshold that maximizes performance on the inner validation set. Next, in the outer loop of the cross-validation, the SLDA weights were computed according to the *ℓ*_1_-norm and *ℓ*_2_-norm regularization hyperparameters selected previously. The parameters of the FastICA, the normalization parameters, the hyperparameters and the parameters of SLDA, as well as the probability threshold were carried over from the training to the test folds of the outer cross-validation loop. For a realistic outcome, joint probability artifact rejection was not applied to the test folds of the outer cross-validation loop. Hence, no trials have been removed from the test folds. Next, on the test folds of the outer loop we assessed the probability output of the detector on all the trials that were executed according to the experimental protocol. In this assessment we denoted the pre-movement class as the positive class. The cross-validation procedure follows the methodology described in [22].

The performance of the detection model was evaluated by the percentage of correctly classified trials for each of the five folds. A trial is correctly classified if it has nonzero sensitivity and maximum specificity. More specifically if it contains at least one true positive window in the ‘pre-movement state’ time interval and has no false positive windows in the ‘relaxation state’ time interval [22].

### Intrinsic Motivation Inventory

To assess the participants’ motivation to perform the experimental task, we used the Intrinsic Motivation Inventory (IMI), which is a multidimensional measurement tool [34] with seven scales (interest/enjoyment, perceived competence, effort/importance, felt pressure/tension, perceived choice, value/usefulness and personal relatedness scale). We used this tool to explore potential correlations between patients’ overall motivation to perform the rehabilitation related task and the accuracy of the BCI-detector of their intention to walk. The questionnaire was administered at the end of each session of the experiment. During this experiment we used only the first six subscales. The interest/enjoyment subscale is considered the self-report measure of intrinsic motivation; thus, although the overall questionnaire is called the Intrinsic Motivation Inventory, it is only the one subscale that assesses intrinsic motivation, per se. The perceived choice and perceived competence concepts are considered positive predictors of both self-report and behavioral measures of intrinsic motivation, and pressure/tension is known as a negative predictor of intrinsic motivation [34]. Effort is a separate variable that is relevant to some motivation questions. The value/usefulness subscale is used for patients to internalize and become self-aware of the activities that they experience as useful or valuable for themselves. The questionnaire used in this experiment had a total of 37 questions. Participants gave scores on a seven-point Likert scale (1: not at all true and 7: very true).

## Results

### Neurophysiological analysis

EEG pre-movement correlates, MRCP (0.1−1 Hz) and *μ* band (8−13 Hz), were used to assess the intention to walk in all 27 cases (9 subjects x 3 sessions). Statistically significant neural correlates were found in 19 cases. For the statistical analysis we used as measurement points the mean of the ‘pre-movement state’ time interval for ten EEG-channels (F3, Fz, F4, FC1, FC2, C3, Cz, C4, CP1 and CP2) for the MRCP and logarithmic power in the *μ* band.

**Fig. 3:**
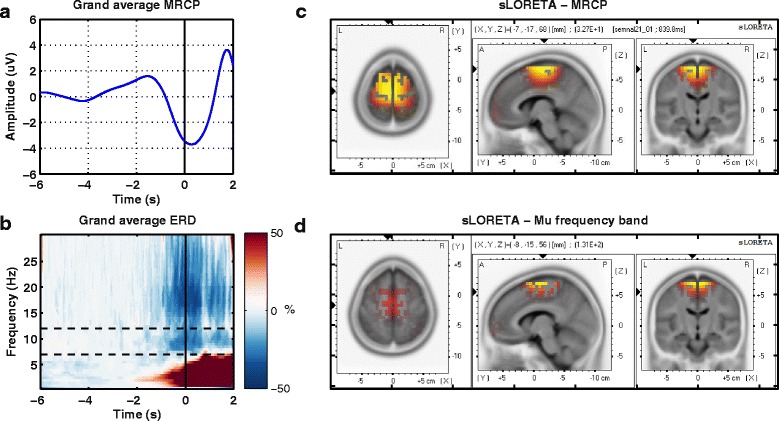
Neurophysiological results. **a**. Grand average MRCP over subjects and sessions for all nineteen cases; **b**. Grand average ERD over subjects and sessions. The dashed line marks the mu frequency band later used in classification. In both figures, the black vertical line represents the onset of motion; **c** and **d**. Source localization results for MRCP temporal features and *μ* band spectral features at movement onset are displayed from three perspectives: top view, lateral view from the left and back view

A two-way ANOVA with factor subject and three repetitions (sessions) was conducted to compare the subjects’ neural correlates. The null hypothesis was rejected as the measurement points were significantly different between subjects (F(2, 8) = 11.56 (9.41), *p*<0.05). Post-hoc comparisons for the MRCP and *μ* band using the Tukey-Kramer critical value indicated that the mean score for subject 1 session 2 and 3, subject 4 session 2, subject 6 session 2, subject 7 sessions 1 and 3, subject 9 sessions 1 and 3 were significantly different than the mean of the other subjects (M =−4.36 (0.87), SD = 0.89 (0.47)). These subjects and sessions were discarded from the grand average analysis (Fig. 3), but were kept for the rest of the analysis, as we are interested in a realistic evaluation of our detection model (see Figure 8 from Appendix).

Figure 3 shows the grand average processes. Figure 3a presents the MRCP correlate as a negative amplitude deflection starting a second and a half before the onset and reaching maximum negativity 200 ms after the movement onset. Figure 3b shows the time-frequency representation, in the frequency range of 0.1−30 Hz. The dashed lines mark the *μ* band in which the desynchronization starts a second before the onset and persists during the movement. The brain regions that elicited the most activity during the intention of motion state were Brodmann areas 4 and 6, as shown in Fig. 3c and Fig. 3d.

The individual results for each of the nineteen cases are consistent with the presented results. There was no significant difference (two-sample *t*-test, *p*>0.05, Bonferroni correction) in the neural correlates representation for footedness between subjects.

### Feature selection and classification

**Fig. 4:**
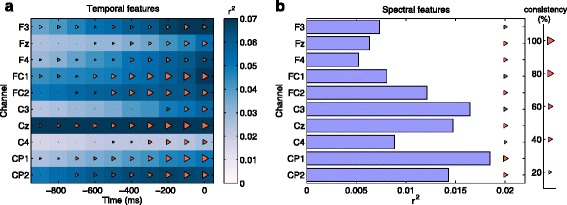
Feature selection. *r* ^2^ values and consistency in feature selection on a time window of one second for the MRCP detection model (**a**) and the ERD detection model (**b**). Consistency stands for how often a feature is selected across all sessions and all subjects and is depicted by the size of the triangle, on a percentage scale from 0 to 100

Figure 4 presents the point-biserial correlation coefficient *r*^2^ between features and classes, and the consistency in feature selection attained by the MRCP and ERD detection models, across all subjects and all recording sessions. The two evaluations are used in a complementary manner. Since *r*^2^ is a univariate measure it reports discriminability at a feature level, while consistency is a multivariate measure derived from the sparse weights of the classifier. Higher discriminability was attained between rest and pre-movement states for the MRCP features than for ERD features, see Fig. 4a, b. More specifically, for the MRCP features in Fig. 4a, central channel Cz, precentral channels FC1 and FC2 and post central channels CP2 were the most discriminative in the last 200 ms window. Figure 4b shows the *r*^2^ values for the *μ* desynchronization power features attained with the ERD detection model. The channels that discriminate rest state from pre-movement state are fronto-central FC2, central C3 and Cz and postcentral CP1 and CP2.

The consistency in feature selection represents how often a feature is selected by the SLDA detection model during all the sessions and across all the subjects. In Fig. 4a the features that were most frequently selected (84 %) belong to the last 200 ms window for the central channel (Cz) and the precentral channels FC1. These channels also presented discriminative *r*^2^ values in the last 200 ms window. Similarly, Fig. 4b shows the *r*^2^ values and the consistency in feature selection for the *μ* desynchronization power features attained with the ERD detection model. The features that were mostly selected (61 %) belong to postcentral channel CP2, and with a 58 % consistency in selection to central channels Cz. The ensemble detection model had the magnitude of the weights ratio of 1/6 in the selection of ERD features to MRCP features.

### Detection model evaluation

**Fig. 5:**
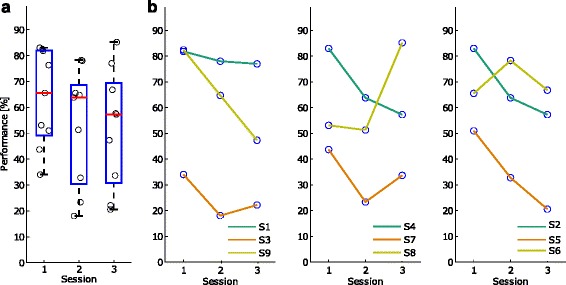
Performance as a percentage of correctly classified trials. Distribution of session-specific performance as a percentage of correctly classified trials for the nine subjects (**a**) and subject-specific transitions in performance across the three sessions (**b**). The circles represent the performance of each subject Performance as a percentage of correctly classified trials. Distribution of session-specific performance as a percentage of correctly classified trials for the nine subjects (first panel from the left) and subject-specific transitions in performance across the three sessions (next panels). The circles represent the performance of each subject

Figure 5 presents the performance as percentage of correctly classified trials during the three sessions. The obtained accuracies were above chance level, which was computed by randomly interchanging the labels of a whole sequence of sliding windows between classes (33 windows for the rest class and 8 windows for the pre-movement class). Due to class imbalance and to a long sequence length (41 windows), the chance level was 4 %.

We found no statistically significant difference between sessions (two-sampled *t*-test, *p*>0.05, Bonferroni correction). In Fig. 5a, the first session has the largest median value of 66 %, while the second session has a median performance of 64 %, followed by the third session with 57 % median performance. More specifically, Fig. 5b presents for every three subjects the transition in performance between the three sessions. Subjects 2, 4, 5, 8 and 9 show large variability in performance between sessions.

**Fig. 6:**
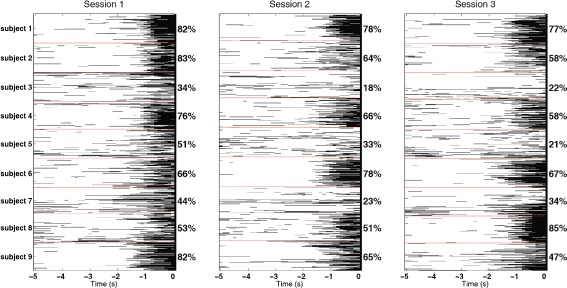
Single-trial chronologically ordered classification outputs for each subject during the three sessions. The percentages represent the average performance of all the test folds in the cross-validation. Red lines are used to separate the subjects within a session. The black color is associated with the pre-movement class and the white one with the rest class

Figure 6 shows the behavior of the detection model after applying the probability threshold and obtaining a binary output. We present single-trial outputs chronologically, for each subject and each session in an interval of five seconds prior to the movement. During the first session our method achieved for subject 3 and subject 7 the lowest detection rate, 34 % and 44 % respectively. These two subjects, had particular medical conditions (brain surgery and titanium plate over the right brain hemisphere). Moreover, subject 7 was also found significantly different from the rest of the group during the neurophysiological analysis. In the following sessions for these two subjects we also attained lower performances (18 %, 23 %, during session 2, and 22 %, 34 %, during session 3, respectively). For subject 5 we found poor scores in the second and third sessions (33 % and 21 %).

### Intrinsic Motivation Inventory results

**Fig. 7:**
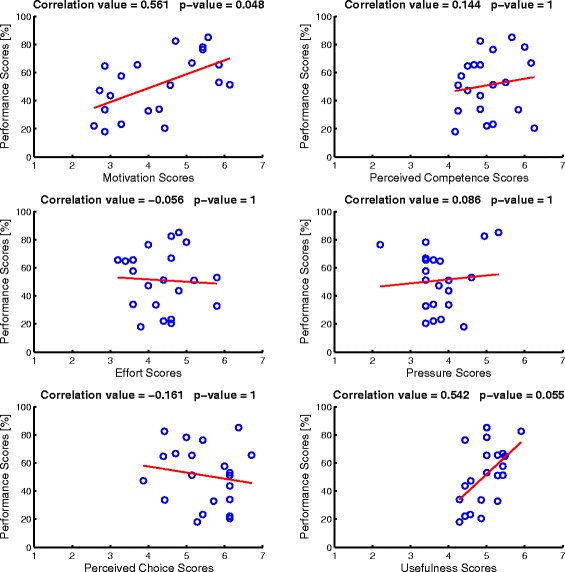
Correlation measurements between performance and psychological factors. The psychological factors are motivation (top left panel), perceived competence (top right panel), effort (middle left panel), pressure (middle right panel), perceived choice (bottom left panel), and usefulness (bottom right panel). The red line shows the linear-regression for each distribution

Figure 7 shows the correlation between the performance scores and the psychological results related to the task. These factors are motivation, perceived competence, effort, pressure, perceived choice and usefulness. The psychological results were collected using the Intrinsic Motivation Inventory [34]. Figure 7 presents the measurements for the last seven subjects during the three sessions, because the first two subjects have not filled out the questionnaire. On the top of each panel is shown the correlation score and the significance *p*-value of each correlation measurement. The correlation between performance and motivation (*r*= 0.561), as well as the correlation between performance and usefulness (*r*= 0.542) are the only ones that have a significant and close to significant *p*-value (*p*= 0.048 for motivation and *p*= 0.055 for usefulness) after Bonferroni-Holm correction [35].

## Discussion

In the present work we show, first, that stroke EEG data related to pre-walking state is classifiable using a detector that combines temporal and spectral features. Our detection model is based on a linear discriminant classifier that combines the outputs of MRCP-based and ERD-based sparse classifiers. For the evaluation of the detection model, we used the percentage of correctly classified trials as a metric of performance [22]. We attained a median detection rate of 64 % in a range between 18 % and 85.2 %, whereas the chance level was 4 %.

Second, based on previous findings [36], we hypothesized that for patients highly motivated to execute the rehabilitation related tasks we will attain higher detection rates. Therefore, we assessed the intrinsic motivation [34] to perform therapy related sessions, and found that the self-reported motivation and the perceived usefulness of the rehabilitation task had a strong positive effect on the number of correct detections of walking intention.

### Neurophysiological findings

At a neurophysiological level, we assessed the difference over the three sessions (repetitions) between subjects at the beginning of the analysis by a statistical measure (two-way ANOVA) using the patients’ neural correlates (MRCP and ERD) as dependent variables. After correcting for multiple comparisons, we found that some of the samples corresponding to a certain subject and session (e.g. subject 1 session 3) had measurement points far from the common mean and were rejected from the electrophysiological analysis. The variability of the MRCP and ERD is shown in Figure 8 from Appendix. The difference in the neural correlates can be seen both between sessions and subjects. For example, subject 2 and subject 8 have different session-specific and subject-specific patterns.

Even though a direct comparison is not straightforward due to several differences, mainly in age and medical condition, the similarity of the grand average EEG-based pre-movement correlates can be observed between healthy young adults [22] and chronic stroke patients. However, it remains an open question whether similar results could be obtained with subacute stroke patients. Patients in an earlier stage (acute or subacute) of the stroke could benefit more from a self-paced and self-initiated rehabilitation therapy.

### BCI detection of pre-movement state

EEG-based BCIs have been extensively used in studies about the detection of upper-limbs movement intention in healthy subjects [12, 37–40], and in stroke patients [10, 12, 41, 42]. In spite of the large number of stroke incidents affecting the lower limbs and the gait function, less attention has been devoted to studies about the detection of lower-limb pre-movement state in healthy subjects [21–23, 43], and even less in stroke patients [10, 12, 41, 42].

Furthermore, the majority of existing EEG studies on pre-movement state detection use either low-frequency amplitude features [12, 23, 37, 44, 45] or spectral features for the analysis [10, 46–49]. Recent studies on healthy subjects report the usage of MRCP features to detect the intention to move the lower-limbs, in tasks such as foot torque movements [43], ankle dorsiflexions [23] or gait movements [21, 22]. For the detection of pre-movement state in foot torque movements [43] the accuracy ranged between 60 % and 84.2 % depending of the family of wavelets used in classification. In the case of ankle-dorsiflexions pre-movement state detection [23] an accuracy of 82.5 ± 7.8 % (*N* = 15) has been obtained using an optimized spatial filtering technique.

The detection of gait pre-movement state has been presented in [21, 22]. In [21] a true positive rate (TPR) detection of 76.9 % and a false positive rate (FPR) of 2.93 ± 1.09 per minute has been reported using a template-matching technique based on one second long windows prior to the MRCP peak. The detection was performed until one second after the motion onset. In a previous study [22] we evaluated the detection of gait intention until motion onset and found a TPR of 67.2 ± 5.9 % and a FPR of 0.79 ± 0.17 per minute, using an asynchronous detector that combines MRCP and ERD features. In the current study on chronic stroke patients, we obtained a lower median performance (64 %), as percentage of correctly classified trials, compared to the results reported previously in healthy subjects (70 % correctly classified trials). Moreover, we showed in [22] that the transfer between sessions, more precisely the removal of session-specific calibration of the BCI detector, introduces a decay in performance of 4 % for intervals of one or two weeks between sessions. For the chronic stroke patients in this study, the intersession transfer performance was lower than 30 %. The attained decay in performance was more than 32 % compared to the previous decay of only 4 %, in healthy young adults.

The lower signal-to-noise ratio in the stroke patients, relative to the one observed in healthy subjects [22], could explain the larger decay in performance between sessions. Moreover, this difference in the signal-to-noise ratio between the two groups of subjects could be due to several factors such as age, brain condition, medication, fatigue, and others, that lead to variability in the neural correlates. During the recovery period, patients in earlier stroke stages (acute and subacute) have shown large variability in their brain activity [50–53]. However, in our study the patients were in later stages of chronic stroke (between 1.4 and 16 years following stroke) and it is unlikely that the session-to-session decay is due to the recovery and to the associated brain plasticity, however further investigation is needed.

Nevertheless a direct comparison with previous studies is not easy due to difference in experimental protocol and methodology. One should note that the metric we use to quantify the performance in [22] and in the current study is the number of correctly classified trials. This metric applies a highly restrictive criterion since it considers a trial as correctly detected only if it has non-zero sensitivity (at least one window correctly classified as pre-movement state) and maximum specificity (no false positive detections). This metric is suitable for rehabilitation scenarios where the number of false positive detections (e.g. activations of a prosthetic device) should be minimized.

There are few studies that report the usage of EEG signals for the detection of intention to move the lower-limbs in stroke patients. One recent study [23] shows the detection of overt and covert self-paced execution of ankle-dorsiflexion in both healthy subjects and stroke patients. The data was analyzed with different spatial filters and a matched filter supervised approach to determine the detection accuracy and latency of MRCP. They attained a TPR of 55.01 ± 12.01 % for stroke patients (*N* = 5). In another study [24], a functional electrical stimulation (FES) rehabilitation device was triggered by the intention to perform ankle-dorsiflexion of one stroke patient. The intention to move was decoded from ERD using an EEG-based BCI. They evaluated the feasibility of the rehabilitation technique using ERD-modulated FES in comparison with FES without ERD modulation and found potential improvements of limb function.

### Intrinsic Motivation Inventory outcomes

The Intrinsic Motivation Inventory results show that the self-reported motivation of the patients, as well as their perceived usefulness of the undergoing task are positively correlated with the detection rate of their intention to walk (*r*= 0.561, *r*= 0.542, respectively). However, the other scales did not show any correlation with performance.

Given the promising results of the correlation between motivation and performance, it would be interesting to further explore the role of motivation or other psychological factors, as regressors for neurophysiological data. Such a regression approach would require a larger number of subjects as presented in a recent study [54], which links the BCI performance modulated by sensorimotor rhythms to several psychological measurements.

### Conclusion

In conclusion, we show that an EEG-based detector of intention to walk can be used in stroke patients, attaining a median performance of 64 % within a range of 18 % and 85.2 %, whereas the chance level is 4 %. Moreover, we found a strong positive link between the motivation of the patients to execute the task and the attained detection rate of their intention to walk. Future work could be directed towards different goals. First, on the improvement in performance during session to session transfer. Specifically, the increase in performance on the transfer between sessions leads to the reduction of session-specific calibration time, that is often tiring and demanding for the patients. Another aim could be to test robustness of the proposed model in a gait-rehabilitation scenario, allowing to close the loop in which the user naturally utilizes his neural signals to activate a prosthetic device.

## Appendix

### Grand average MRCP and ERD for each subject over selected sessions

**Fig. 8:**
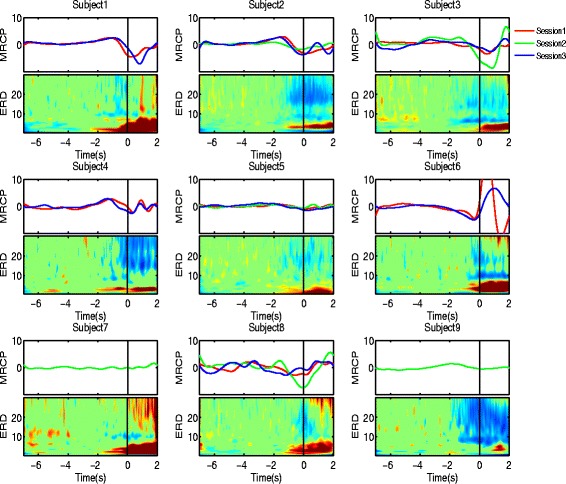
Grand average MRCP (upper subplots) and ERD (lower subplots) on channel Cz over selected sessions for each of the nine subjects. In the upper subplots the ordering of the sessions is marked by the RGB color code (red - session 1, green - session 2, blue - session 3). On the lower subplots, the time-frequency representation shows the ERD across the range of frequencies from 0.1 to 30 Hz. The black vertical line corresponds to the onset of motion

## Endnote

^1^ This project is part of the Spanish Ministry of Science Consolider Ingenio program, HYPER (Hybrid Neuroprosthetic and Neurorobotic Devices for Functional Compensation and Rehabilitation of Motor Disorders) - CSD2009-00067.

## References

[1] Wijdicks E, Sheth K, Carter B, Greer D, Kasner S, Kimberly W (2014). American heart association stroke council. Recommendations for the management of cerebral and cerebellar infarction with swelling: a statement for healthcare professionals from the American Heart Association/American Stroke Association. Stroke.

[2] Latham NK, Jette DU, Slavin M, Richards LG, Procino A, Smout RJ (2005). Physical therapy during stroke rehabilitation for people with different walking abilities. Arch Phys Med Rehabil.

[3] Daly JJ, Wolpaw JR (2008). Brain–computer interfaces in neurological rehabilitation. Lancet Neurol.

[4] Belda-Lois JM, Mena-del Horno S, Bermejo-Bosch I, Moreno JC, Pons JL, Farina D (2011). Rehabilitation of gait after stroke: a review towards a top-down approach. J Neuroeng Rehabil.

[5] Ramos-Murguialday A, Broetz D, Rea M, Läer L, Yilmaz Ö, Brasil FL (2013). Brain–machine interface in chronic stroke rehabilitation: a controlled study. Ann Neurol.

[6] Buch E, Weber C, Cohen LG, Braun C, Dimyan MA, Ard T (2008). Think to move: a neuromagnetic brain-computer interface (bci) system for chronic stroke. Stroke.

[7] Kornhuber HH, Deecke L (1965). Changes in the brain potential in voluntary movements and passive movements in man: readiness potential and reafferent potentials. Pflugers Archiv fur die gesamte Physiologie des Menschen und der Tiere.

[8] Shibasaki H, Hallett M (2006). What is the bereitschaftspotential?. Clinical Neurophysiology.

[9] Pfurtscheller G, Aranibar A (1979). Evaluation of event-related desynchronization (erd) preceding and following voluntary self-paced movement. Electroencephalogr Clin Neurophysiol.

[10] Antelis JM, Montesano L, Ramos-Murguialday A, Birbaumer N, Minguez J. Continuous decoding of intention to move from contralesional hemisphere brain oscillations in severely affected chronic stroke patients. In: Engineering in Medicine and Biology Society (EMBC), 2012 Annual International Conference of the IEEE. IEEE: 2012. p. 4099–103. 10.1109/EMBC.2012.6346868 23366829

[11] López-Larraz E, Antelis JM, Montesano L, Gil-Agudo A, Minguez J. Continuous decoding of motor attempt and motor imagery from eeg activity in spinal cord injury patients. In: Engineering in Medicine and Biology Society (EMBC), 2012 Annual International Conference of the IEEE. IEEE: 2012. p. 1798–1801. 10.1109/EMBC.2012.6346299 23366260

[12] Lew E, Chavarriaga R, Silvoni S, Millán JdR. Detection of self-paced reaching movement intention from eeg signals. Front Neuroengineering. 2012;5(13). 10.3389/fneng.2012.00013 PMC3458432 23055968

[13] Green JB, Bialy Y, Sora E, Ricamato A (1999). High-resolution eeg in poststroke hemiparesis can identify ipsilateral generators during motor tasks. Stroke.

[14] Sandhya B, Shendkar C, Mahadevappa M. Single channel event related (de) synchronization (erd/ers) analysis of motor execution in stroke affected foot drop subjects. In: Medical Imaging, m-Health and Emerging Communication Systems (MedCom), 2014 International Conference On. IEEE: 2014. p. 325–8.

[15] Wheaton LA, Mizelle J, Forrester LW, Bai O, Shibasaki H, Macko RF (2007). How does the brain respond to unimodal and bimodal sensory demand in movement of the lower extremity?. Exp Brain Res.

[16] Solis-Escalante T, Müller-Putz G, Pfurtscheller G (2008). Overt foot movement detection in one single laplacian eeg derivation. J Neurosci Methods.

[17] Bulea TC, Prasad S, Kilicarslan A, Contreras-Vidal JL. Sitting and standing intention can be decoded from scalp eeg recorded prior to movement execution. Front Neurosci. 2014;8(376). 10.3389/fnins.2014.00376 PMC4243562 25505377

[18] Wagner J, Solis-Escalante T, Grieshofer P, Neuper C, Müller-Putz G, Scherer R (2012). Level of participation in robotic-assisted treadmill walking modulates midline sensorimotor eeg rhythms in able-bodied subjects. Neuroimage.

[19] Velu PD, de Sa VR. Single-trial classification of gait and point movement preparation from human eeg. Front Neurosci. 2013;7(84). 10.3389/fnins.2013.00084 PMC3678086 23781166

[20] Presacco A, Forrester L, Contreras-Vidal JL. Towards a non-invasive brain-machine interface system to restore gait function in humans. In: Engineering in Medicine and Biology Society, EMBC, 2011 Annual International Conference of the IEEE. IEEE: 2011. p. 4588–591. 10.1109/IEMBS.2011.6091136 22255359

[21] Jiang N, Gizzi L, Mrachacz-Kersting N, Dremstrup K, Farina D (2015). A brain–computer interface for single-trial detection of gait initiation from movement related cortical potentials. Clin Neurophysiol.

[22] Sburlea AI, Montesano L, Minguez J (2015). Continuous detection of the self-initiated walking pre-movement state from eeg correlates without session-to-session recalibration. J Neural Eng.

[23] Niazi IK, Jiang N, Tiberghien O, Nielsen JF, Dremstrup K, Farina D (2011). Detection of movement intention from single-trial movement-related cortical potentials. J Neural Eng.

[24] Takahashi M, Takeda K, Otaka Y, Osu R, Hanakawa T, Gouko M (2012). Event related desynchronization-modulated functional electrical stimulation system for stroke rehabilitation: A feasibility study. J Neuroeng Rehabil.

[25] Elias LJ, Bryden MP, Bulman-Fleming MB (1998). Footedness is a better predictor than is handedness of emotional lateralization. Neuropsychologia.

[26] Winter D, Yack H (1987). Emg profiles during normal human walking: stride-to-stride and inter-subject variability. Electroencephalogr Clin Neurophysiol.

[27] Delorme A, Makeig S (2004). Eeglab: an open source toolbox for analysis of single-trial eeg dynamics including independent component analysis. J Neurosci Methods.

[28] Bingham E, Hyvärinen A (2000). A fast fixed-point algorithm for independent component analysis of complex valued signals. Int J Neural Syst.

[29] Tallon-Baudry C, Bertrand O (1999). Oscillatory gamma activity in humans and its role in object representation. Trends Cogn Sci.

[30] Graimann B, Pfurtscheller G (2006). Quantification and visualization of event-related changes in oscillatory brain activity in the time–frequency domain. Prog Brain Res.

[31] Pascual-Marqui RD (2002). Standardized low-resolution brain electromagnetic tomography (sloreta): technical details. Methods Find Exp Clin Pharmacol.

[32] Clemmensen L, Hastie T, Witten D, Ersbøll B (2011). Sparse discriminant analysis. Technometrics.

[33] Lemm S, Blankertz B, Dickhaus T, Müller KR (2011). Introduction to machine learning for brain imaging. Neuroimage.

[34] Ryan RM, Deci EL (2000). Self-determination theory and the facilitation of intrinsic motivation, social development, and well-being. Am Psychol.

[35] Holm S (1979). A simple sequentially rejective multiple test procedure. Scand J Stat.

[36] Nijboer F, Birbaumer N, Kübler A. The influence of psychological state and motivation on brain–computer interface performance in patients with amyotrophic lateral sclerosis–a longitudinal study. Front Neurosci. 2010;4(55). 10.3389/fnins.2010.00055 PMC2916671 20700521

[37] Garipelli G, Chavarriaga R, del R Millán J (2013). Single trial analysis of slow cortical potentials: a study on anticipation related potentials. J Neural Eng.

[38] Bai O, Rathi V, Lin P, Huang D, Battapady H, Fei DY (2011). Prediction of human voluntary movement before it occurs. Clin Neurophysiol.

[39] López-Larraz E, Montesano L, Gil-Agudo Á, Minguez J (2014). Continuous decoding of movement intention of upper limb self-initiated analytic movements from pre-movement eeg correlates. J Neuroeng Rehabil.

[40] Ahmadian P, Sanei S, Ascari L, González-Villanueva L, Umilta MA (2013). Constrained blind source extraction of readiness potentials from eeg. IEEE Trans Neural Syst Rehabil Eng.

[41] Ibáñez J, Serrano JI, del Castillo MD, Barrios L, Gallego JÁ, Rocon E. An eeg-based design for the online detection of movement intention. In: Advances in Computational Intelligence. Berlin Heidelberg: Springer. 6691;2011:370–7.

[42] Ibáñez J, Serrano JI, del Castillo MD, Monge E, Molina F, Rivas F, et al. Detection of the onset of voluntary movements based on the combination of erd and bp cortical patterns. In: Replace, Repair, Restore, Relieve–Bridging Clinical and Engineering Solutions in Neurorehabilitation. Springer: 2014. p. 437–46.

[43] Farina D, Nascimento OFd, Lucas MF, Doncarli C (2007). Optimization of wavelets for classification of movement-related cortical potentials generated by variation of force-related parameters. J Neurosci Methods.

[44] Do Nascimento OF, Nielsen KD, Voigt M (2006). Movement-related parameters modulate cortical activity during imaginary isometric plantar-flexions. Exp Brain Res.

[45] Niazi IK, Jiang N, Jochumsen M, Nielsen JF, Dremstrup K, Farina D (2013). Detection of movement-related cortical potentials based on subject-independent training. Med Biol Eng Comput.

[46] Nam CS, Jeon Y, Kim YJ, Lee I, Park K (2011). Movement imagery-related lateralization of event-related (de) synchronization (erd/ers): Motor-imagery duration effects. Clin Neurophysiol.

[47] Neuper C, Pfurtscheller G (2001). Event-related dynamics of cortical rhythms: frequency-specific features and functional correlates. Int J Psychophysiol.

[48] Severens M, Nienhuis B, Desain P, Duysens J. Feasibility of measuring event related desynchronization with electroencephalography during walking. In: Engineering in Medicine and Biology Society (EMBC), 2012 Annual International Conference of the IEEE. IEEE: 2012. p. 2764–767. 10.1109/EMBC.2012.6346537 23366498

[49] Müller-Putz GR, Zimmermann D, Graimann B, Nestinger K, Korisek G, Pfurtscheller G (2007). Event-related beta eeg-changes during passive and attempted foot movements in paraplegic patients. Brain Res.

[50] Dam M, Tonin P, Casson S, Ermani M, Pizzolato G, Iaia V (1993). The effects of long-term rehabilitation therapy on poststroke hemiplegic patients. Stroke.

[51] Ferrucci L, Bandinelli S, Guralnik J, Lamponi M, Bertini C, Falchini M (1993). Recovery of functional status after stroke. A postrehabilitation follow-up study. Stroke.

[52] Giaquinto S, Cobianchi A, Macera F, Nolfe G (1994). Eeg recordings in the course of recovery from stroke. Stroke.

[53] Tangwiriyasakul C, Verhagen R, Rutten WL, van Putten MJ (2014). Temporal evolution of event-related desynchronization in acute stroke: a pilot study. Clin Neurophysiol.

[54] Hammer EM, Halder S, Blankertz B, Sannelli C, Dickhaus T, Kleih S (2012). Psychological predictors of smr-bci performance. Biol Psychol.

